# Current Limitations and Novel Perspectives in Pancreatic Cancer Treatment

**DOI:** 10.3390/cancers14040985

**Published:** 2022-02-16

**Authors:** Steve Robatel, Mirjam Schenk

**Affiliations:** Institute of Pathology, Experimental Pathology, University of Bern, 3008 Bern, Switzerland; steve.robatel@pathology.unibe.ch

**Keywords:** PDAC, TME, cancer therapy, pancreatic cancer

## Abstract

**Simple Summary:**

This review article presents a synopsis of the key clinical developments, their limitations, and future perspectives in the treatment of pancreatic cancer. In the first part, we summarize the available treatments for pancreatic cancer patients according to tumor stage, as well as the most relevant clinical trials over the past two decades. Despite this progress, there is still much to be improved in terms of patient survival. Therefore, in the second part, we consider various components of the tumor microenvironment in pancreatic cancer, looking for the key drivers of therapy resistance and tumor progression, which may lead to the discovery of new potential targets. We also discuss the most prominent molecules targeting the stroma and immune compartment that are being investigated in either preclinical or clinical trials. Finally, we also outline interesting venues for further research, such as possible combinations of therapies that may have the potential for clinical application.

**Abstract:**

Pancreatic cancer is one of the deadliest cancers worldwide, largely due to its aggressive development. Consequently, treatment options are often palliative, as only one-fifth of patients present with potentially curable tumors. The only available treatment with curative intent is surgery followed by adjuvant chemotherapy. However, even for patients that are eligible for surgery, the 5-year OS remains below 10%. Hence, there is an urgent need to find new therapeutic regimens. In the first part of this review, we discuss the tumor staging method and its impact on the corresponding current standard-of-care treatments for PDAC. We also consider the key clinical trials over the last 20 years that have improved patient survival. In the second part, we provide an overview of the major components and cell types involved in PDAC, as well as their respective roles and interactions with each other. A deeper knowledge of the interactions taking place in the TME may lead to the discovery of potential new therapeutic targets. Finally, we discuss promising treatment strategies targeting specific components of the TME and potential combinations thereof. Overall, this review provides an overview of the current challenges and future perspectives in the treatment of pancreatic cancer.

## 1. Introduction

Pancreatic cancer, in its most common form—pancreatic ductal adenocarcinoma (PDAC)—is one of the most lethal cancers worldwide. PDAC is the third leading cause of cancer death and is expected to become the first within the next ten years. With its five-year survival rate of 7%, PDAC is characterized by its aggressive nature and rapid metastasis formation. Moreover, the symptoms of PDAC, such as back pain, loss of appetite, weight loss and new-onset diabetes [1], are not specific and are often misinterpreted, leading to a late diagnosis [2]. In fact, less than 20% of PDAC patients present local and potentially curable tumors. While the cause of pancreatic cancer remains unknown, some factors such as smoking [3], chronic pancreatitis, which can be caused by excessive alcohol consumption [4,5], and age have been associated with an increased risk for the development of PDAC [6,7]. Furthermore, some pathogenic germline gene variants are known to increase susceptibility to PDAC, mainly occurring in DNA damage repair genes. The most observed variants in PDAC include *BRCA1/2* (breast cancer gene 1/2) and *ATM* (ataxia telangiectasia mutated).

In most cases, pancreatic cancer originates in the ductal epithelium and develops from pre-malignant lesions. The best-characterized pre-malignant lesion and precursor of pancreatic cancer is called pancreatic intraepithelial neoplasia (PanIN) [8]. As cancer develops, the minimally dysplastic epithelium, called pancreatic intraepithelial neoplasia grades 1A and 1B, progresses to more severe dysplasia called pancreatic intraepithelial neoplasia grades 2 and 3 to finally reach the stage of an invasive carcinoma. In parallel, successive mutations accumulate, including the activation of the *KRAS* oncogene, the inactivation of the tumor-suppressor gene *CDKN2A*, and finally the inactivation of the tumor-suppressor gene *TP53* and deleted in pancreatic cancer 4 (*DPC4)*, also known as the SMAD family member 4 (*SMAD4*). The inactivation of *CDKN2A* results in the loss of the p16 protein, a regulator of the G1-S transition of the cell cycle, and the activation of TP53 allows cells to bypass DNA damage control checkpoints. The mutation of *SMAD4* results in aberrant transforming growth factor β (TGF-β) signaling. Other less well characterized premalignant lesions of the pancreas have been described and characterized as intrapancreatic mucinous neoplasia (IPMN) and mucinous cystic neoplasia (MCN) [9]. The Genome Project showed that PDAC is a type of tumor with high inter-tumoral genetic heterogeneity [10]. This suggests that it might not be possible to find a single therapy for all PDAC patients. In fact, two major molecular subtypes of PDAC have recently been described, namely the classical and the basal-like subtypes. The classical subtype is characterized by a higher differentiation of the tumor, fibrosis, and inflammation, while the basal-like subtype shows a loss of differentiation and is associated with poor survival and a lack of response to existing chemotherapy regimens [11]. These findings support the fact that PDAC presents a high inter-patient variability and that treatment regimens may have to be adapted to the different tumor subtypes.

In this review, we will consider the standard-of-care treatments for PDAC and discuss the main features of the TME to find new potential therapeutic targets or combinations thereof. We will also review the most promising clinical trials and relevant questions they raise.

## 2. PDAC Standard-of-Care Treatments

To optimally treat PDAC patients, tumor staging is a crucial step. The staging of pancreatic cancer is based on the results of helical CT and tumor-node-metastasis classification according to the most recent edition of the ‘American Joint Committee on Cancer’ [12] (Table 1). The goal of CT imaging is to determine the position and size of the tumor, the involvement of veins and arteries, and the presence of metastasis. In brief, stages I and II comprise tumors that are limited to the pancreas and have minimal or no contact with major vessels. The difference between stage I and II is the size of the tumor (≤2 cm for I and >2 cm for II). Tumors of stage III are extended beyond the pancreas, involving the superior mesenteric vein, portal vein or splenic vein, but do not involve the celiac axis or the superior mesenteric artery. These three tumor stages can include regional lymph node metastasis. Stage IV tumors involve the superior mesenteric artery or celiac axis [13]. The tumor stage is a benchmark for assessing resectability. Stage I and II are considered resectable, stage II-III tumors are borderline resectable (BRPC) or locally advanced PDAC (LAPC), depending on whether a safe and complete resection and the reconstruction of the affected veins and arteries are possible. In general, the borderline resectable status indicates that the tumor is neither clearly resectable nor clearly unresectable but rather involves a greater likelihood of incomplete resection, including R1 resection and a positive margin, as part of upfront surgery [14]. Finally, stage IV tumors are metastatic PDAC and are considered non-resectable [15]. Precise tumor staging allows clinicians to provide the optimal treatment to the patients based on the advancement of the disease. Within the next sections, we will review the recommended treatments depending on the tumor stage.

### 2.1. Surgery

Today, surgery remains the only cure for patients with pancreatic cancer, but less than 20% of them are eligible for surgery. The main goal of surgery is to resect the entire tumor, in a way that no cancer cells can been seen microscopically at the primary tumor site. This successful resection is termed R0. On the other hand, a resection is called R1 when cancer cells remain microscopically visible at the primary tumor site. Unfortunately, a recent analysis concluded that the proportion of R1 resection exceeds 75%, even in specialized surgical centers [16]. The Whipple procedure, the resection of pancreatic head adenocarcinoma, consists of pancreatoduodenectomy, which includes the resection of the pancreatic head, duodenum, distal common bile, and sometimes gastric antrum, followed by pancreatoenterostomy, hepaticojejunostomy and gastrojejunostomy. This procedure is associated with a high morbidity rate of up to 45%. When the tumor is found in the tail or the body of the pancreas, the performed surgery is termed distal pancreatectomy, where the tail and possibly a part of the body of the pancreas is resected. As a safety measure due to proximity, the spleen is often removed as well. Finally, if the cancer has spread throughout the pancreas but is still resectable, a total pancreatectomy is performed, whereby the entire pancreas is removed, as well as the gallbladder, the spleen and parts of the stomach and the small intestine. This type of resection is the most drastic and is avoided as much as possible due to major side effects, such as diabetes and the inability to digest certain foods. Therefore, patients that have undergone total pancreatectomy are entirely dependent on insulin shots and must take pancreatic enzymes for life.

### 2.2. Neoadjuvant Chemotherapy

Neoadjuvant therapy can eradicate metastases that are not visible with imaging methods and in some cases, tumor shrinkage has been observed, which improves the resectability [17]. However, the benefit of neoadjuvant therapy compared to upfront surgery and adjuvant therapy for resectable PDAC is undergoing evaluation. This is addressed in the ongoing phase III trial (A021806) where perioperative FOLFIRINOX (Ca^2+^ folinate, 5-FU, irinotecan and oxaliplatin) is compared to adjuvant FOLFIRINOX for patients with resectable PDAC [18]. For BRPC tumors, surgery is often an option but is likely to result in R1 resection, which is an important negative prognostic factor for overall survival (OS) [19]. In these cases, neoadjuvant chemotherapy, such as FOLFIRINOX, may increase the rate of successful R0 resection and improve long-term survival [20]. Although neoadjuvant treatment is recommended by the *National Comprehensive Cancer Network* (NCCN), it has not yet become the standard-of-care treatment due to the lack of randomized studies and the added complexity to the multidisciplinary treatment planning. Neoadjuvant therapy requires a pretreatment biopsy and endoscopic stent placement in patients with biliary obstruction. However, a meta-analysis that included 13 trials demonstrated the down-staging of patients with BRPC or unresectable tumors after neoadjuvant FOLFIRINOX therapy, with an R0 resection rate of 40% [21]. Another meta-analysis highlighted the benefits of neoadjuvant therapy in resectable and borderline resectable pancreatic cancer, by comparing survival by intention to treat between patients undergoing upfront surgery and neoadjuvant chemotherapy. A total of 3484 patients were included in the analysis and approximately 50% of them received neoadjuvant treatment. Despite a lower resection rate (66% vs. 81.3%), patients who received neoadjuvant therapy survived longer compared to upfront surgery (18.8 vs. 14.8 months, respectively). The difference was even larger among patients who underwent resection (26.1 vs. 15 months) [22].

### 2.3. Adjuvant Chemotherapy for Resected Pancreatic Cancer

The randomized trial of the ESPAC-1 demonstrated that adjuvant fluorouracil (5-FU) chemotherapy has a beneficial impact on the OS of the patients after R0 and R1 resection (Table 2) [23]. The phase III CONKO-001 trial showed similar positive results after gemcitabine treatment, with prolonged disease-free survival (DFS) and OS, including in patients with R0- and R1-resected tumors (Table 2). Long-term follow up also showed an increased 10-year OS of 5% [24,25]. Finally, the phase III ESPAC-3 trial compared adjuvant therapy with either gemcitabine or 5-FU plus folinic acid and concluded that there was no significant difference between the two options [26] (Table 2). Therefore, both are currently considered standard treatments [15]. The benefit of adjuvant radiotherapy remains controversial. Therefore, treatment with systemic chemotherapy has become the standard of care after R0 and R1 resection. Recently, impressive progress has been achieved with FOLFIRINOX as adjuvant treatment for patients with resected PDAC. As shown in the PRODIGE 24/CCTG PA.6 trial, the median OS of the FOLFIRINOX group reached 53.5 months compared to 35.5 months in the gemcitabine group. A recent update of this trial included the 5-year patient follow up. The 5-year DFS rate is 26.1% for patients treated with FOLFIRINOX vs. 19.0% for gemcitabine, and the 5-year OS rate is 43.2% vs. 31.4%, respectively [27] (Table 2). Although FOLFIRINOX does not cure PDAC, these data demonstrate its positive impact on patient survival and are another step in the right direction. Therefore, FOLFIRINOX is recommended as adjuvant treatment for patients with excellent health status. For patients with poorer general health status, gemcitabine remains the preferred option.

### 2.4. Treatment of Locally Advanced and Metastatic Disease

LAPC is inoperable and up to 80% of the patients will not have sufficient tumor response to neoadjuvant chemotherapy to become eligible for resection. The standard treatment aims to control the disease with chemotherapy, commonly FOLFIRINOX, nab-paclitaxel and/or gemcitabine [28], however, the OS of LAPC patients remains below one year [29]. Clinical trials have shown the equivalent response and efficacy of nab-paclitaxel and gemcitabine or FOLFIRINOX [30,31]. These are therefore considered as standard-of-care treatments and the use of either one is adapted based on the degree of adverse effects observed in each patient [32]. However, for approximately 20% of the patients who respond well to chemotherapy combinations, such as FOLFIRINOX or gemcitabine plus nab-paclitaxel, some studies reported promising results, namely a reduction in tumor size, converting some LAPC into resectable tumors [21].

Chemotherapy remains the standard treatment for metastatic pancreatic cancer. In fact, gemcitabine has been the standard of care for first-line treatment for the past 14 years, as a study has shown its clinical benefits in improving median progression-free survival (PFS). Unfortunately, even with gemcitabine treatment, the OS of patients with metastatic disease is only between five and six months with a response rate of 5.4% [33]. Various combinations of gemcitabine-based treatments with either cytotoxic or molecularly targeted agents have been tested and showed only a slight increase in the OS, from a few weeks to a few months [30,33,34,35,36]. Similarly, several meta-analysis of randomized controlled trials have shown that combining gemcitabine with fluoropyrimidine or platinum compounds moderately improves OS in patients with advanced PDAC [37,38,39]. In the PRODIGE trial of 2011, an important breakthrough for patients with metastatic PDAC was achieved with FOLFIRINOX treatment. The OS of patients treated with FOLFIRINOX increased up to 11.1 months compared to the 6.8 months observed with gemcitabine alone [35] (Table 2). Since this trial, FOLFIRINOX has become the standard of care for patients with metastatic PDAC. This major discovery then led to the use of FOLFIRINOX as adjuvant and neoadjuvant therapy. Recently, Shelemey et al. reported the case of a 59-year-old woman with adenocarcinoma of the pancreatic tail and innumerable liver metastases who received FOLFIRINOX chemotherapy. Subsequent CT scans showed shrinkage of the pancreatic mass, as well as the liver metastases. Her cancer antigen 19-9 (CA 19-9) normalized after 11 months. Oxaliplatin was then discontinued due to peripheral neuropathy, but she still completed 37 cycles of FOLFIRI (Ca^2+^ folinate, 5-FU, irinotecan). During these cycles, the pancreatic mass disappeared, and the liver metastases decreased in size and remained as scar tissue. After 5.5 years, an MRI of her abdomen showed no recurrence of pancreatic mass, and two residual liver lesions, defined as scar tissue, remained stable [40]. This case of complete response to FOLFIRINOX/FOLFIRI chemotherapy is encouraging and argues for its use as standard therapy.

## 3. PDAC TME, Immunotherapies and Therapy Resistance: What to Target Next?

**Figure 1:**
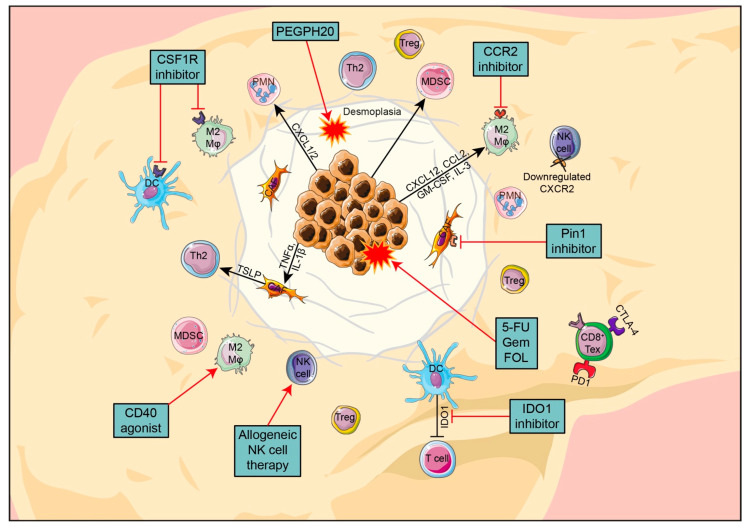
Overview of the key components in PDAC TME, their interactions and promising new targets.

After reviewing the currently available treatments based on tumor stage, we now discuss the major features of PDAC and its TME. In the next section, we will consider possible causes of treatment resistance and how to identify potential new therapeutic targets based on what is known about the TME in PDAC (Figure 1).

### 3.1. Pancreatic Cancer Cells

During the development of PDAC, pancreatic cancer cells acquire several features that enable them to evade the immune system. First of all, cancer cells are able to downregulate their expression of major histocompatibility class I (MHC-I) molecules, making them less well recognized by effector T cells [41,42]. Second, PDAC cells can attract various immunosuppressive cells that further contribute to the low immunogenicity of the tumor. For example, PDAC cells use the CCL2/CCR2 axis to recruit tumor-associated macrophages (TAMs) and myeloid-derived suppressor cells (MDSCs) [43], and the CCL5/CCR5 axis to recruit regulatory T cells (Tregs) [44]. Third, pancreatic cancer cells can reduce effector cell function within the TME by the immune checkpoint molecule programmed death-ligand 1 (PD-L1), which is expressed in approximately 13% of PDAC patients. PD-L1 binds to PD-1 on the T cells and induces their anergy and apoptosis, contributing to immune evasion [45]. Furthermore, PDAC cells produce indoleamine 2,3-dioxygenase (IDO), which catalyzes tryptophan degradation. As tryptophan is required for T cell survival and activation, its degradation also leads to T cell apoptosis and anergy [46]. In addition, it has been shown that IDO enhances the recruitment of Tregs and tryptophan starvation induces their development in the TME [47,48]. Together, these mechanisms allow pancreatic cancer cells to develop and proliferate while being protected from an immune response.

### 3.2. PDAC Stroma

Upon PDAC establishment, one major characteristic is a desmoplastic reaction which is the formation of dense stroma [49]. This stroma is composed of high-density fibrotic tissue and pancreatic stellate cells (PSCs), which are the cancer-associated fibroblasts (CAFs) specific to PDAC and the main feature of PDAC, accounting for nearly 90% of the tumor mass. The formation of a dense stroma is the result of PSCs or myofibroblasts activation by growth factors such as TGF-β1, platelet-derived growth factor (PDGF) and fibroblast growth factor. Activated PSCs secrete collagen and other components of the extracellular matrix which play a role in poor vascularization, which is characteristic of PDAC [50]. However, the role of stromal cells in pancreatic cancer progression seems controversial. In some studies, the presence of stroma has been shown to promote immunosuppression and fibrosis [51,52] and support cancer progression by attenuating antitumor effector mechanisms. The stroma itself increases the number of immunosuppressive cells and inhibits cytotoxic CD8^+^ T cells [53]. In these studies, it seems that the stroma does not only constitute a physical barrier, but also forms a compartment involved in the process of tumor formation, progression, invasion and metastasis [49]. It has been shown that stromal cells express proteins associated with poor prognosis and treatment resistance, such as PDGF receptor, vascular endothelial growth factor (VEGF) and secreted protein, acidic and rich in cysteine (SPARC) [54,55]. However, Özdemir et al. conducted a preclinical study in which CAFs were depleted and surprisingly, they observed that collagen was reduced and matrix was reorganized, angiogenesis was decreased, and hypoxia was enhanced. In addition, the number of cancer stem cells and the frequency of Tregs increased, leading to a poor prognosis [56]. Recently, much progress has been made in deciphering the controversial role of CAFs, which is now known to be due to several contrasting functions. Distinct CAFs populations have been identified that differ in their gene expression and secretome profiles. Myofibroblastic CAFs (myCAFs) are the closest to cancer cells with properties of activated fibroblasts. On the other hand, inflammatory-CAF (iCAFs) are in a more distal location from the tumor and are characterized by the expression of inflammatory mediators, such as IL-1, IL-21, and CXCL1-3 [57,58].

### 3.3. Treatments Targeting PDAC Stroma

The desmoplastic stroma of PDAC not only constitutes a large portion of the tumor mass, but also plays a key role in immunosuppression and acts as a barrier preventing effective therapy. However, treatments targeting the stroma have revealed controversial results, and it is not clear whether the modulation of the stroma is beneficial in PDAC [56,59]. Interestingly, the most clinically advanced stromal modulator, hyaluronidase PEGPH20, has been tested in a phase II trial and has been shown to degrade stromal proteins, improve vascular perfusion, and prolong PFS in combination with standard chemotherapy [60]. Unfortunately, in a Phase III study, PEGPH20 in combination with chemotherapy failed to improve the clinical outcome of PDAC patients [61]. Another stromal modulator and regulator of TME fibrosis and immunosuppression, focal adhesion kinase (FAK), is associated with decreased T cell infiltration when highly expressed in human PDAC. In preclinical studies, the inhibition of FAK enhanced the response to chemotherapy and checkpoint blockade, while reducing the infiltration of MDSCs into the TME [62]. Further studies are required to understand the mechanisms and benefits of targeting the desmoplastic stroma in PDAC.

### 3.4. The Immune Compartment in PDAC

As previously discussed, the TME in PDAC is mainly composed of cancer cell nests and stroma. The stroma itself consists of stromal matrix and immune cells. Immune cells account for up to 50% of the total cell number in PDAC; however, only a small subset of these are tumoricidal cells, whilst the rest are tumor-promoting and immunosuppressive cells [63]. One of the reasons is the release of immunosuppressive cytokines such as IL-10 and TGF-β and the recruitment of immunosuppressive cells during tumorigenesis [64,65]. IL-10 and TGF-β induce Tregs, which also produce IL-10 and TGF-β, creating a positive feedback loop that contributes to the inhibition of effector T cells and the maintenance of immunosuppression [66,67,68]. The immune compartment of PDAC is heterogeneous, the main immune cell types being dendritic cells (DCs), macrophages, neutrophils, MDSCs, natural killer cells (NKs), and effector T cells [69]. In the next section, we will elaborate on the main features of these various cell types, their role in PDAC, and their potential as therapeutic targets.

#### 3.4.1. Dendritic Cells

In PDAC, the immunogenic and pro-inflammatory functions of myeloid cells are largely impaired. DCs have been shown to infiltrate PDAC lesions and their number increases with disease progression from PanIN to PDAC. However, the expression of DC maturation markers, such as MHC class II and the costimulatory molecules CD86 and CD40, is reduced by the action of Tregs, which directly affects CD8^+^ T cell activation and the expansion of tumor-infiltrating DCs [70]. DC function is further impaired by PDAC epithelial cells through the secretion of DC-suppressive cytokines such as IL-10, which downregulate MHC class I and CD40 expression, maintaining DCs in an immature stage [71,72].

#### 3.4.2. Macrophages

In steady-state conditions or upon inflammation, most tissue macrophages originate from bone marrow-derived monocytes in the blood circulation. The remaining portion of macrophages are termed specialized tissue-resident, such as alveolar macrophages in the lungs, microglia in the brain, or Kupffer cells in the liver, and are not derived from blood monocytes [73]. When a tumor develops, it can recruit and induce macrophages in the TME with various chemokines and cytokines, such as CXCL12, CCL2, GM-CSF, colony-stimulating factor 1 (CSF-1), and IL-3. These special macrophages are then called tumor-associated macrophages (TAMs) [74,75]. A recent study showed that certain TAMs may also originate from tissue-resident macrophages, representing a functionally distinct subpopulation [76]. Although they form a continuous spectrum, TAM subpopulations are mainly divided into two opposite polarization states, namely M1 and M2. Typically, M1 macrophages are characterized by the secretion of pro-inflammatory cytokines and a tumoricidal function, while conversely, M2 macrophages secrete anti-inflammatory signals promoting tumor progression [77]. In PDAC, the majority of TAMs display an M2-like phenotype, characterized by the expression of surface markers CD163 and CD206, and the cytokines IL-10 and TGF-β [78]. They are primarily located at the invasive front of the tumor [63,79]. Macrophage infiltration begins early and persists throughout cancer progression [63]. TAM infiltration is correlated with perineural invasion [80], angiogenesis, lymph node metastasis [79,80,81], cancer cell epithelial–mesenchymal transition and extravasation [78]. Therefore, macrophage depletion reduces lung and liver metastasis in an orthotopic mouse model of PDAC [43]. Thus, TAMs appear to be involved in both regulating PDAC invasion and metastasis and are therefore correlated with worse OS. Finally, TAMs are partially responsible for the impaired efficacy of chemotherapy in PDAC. It has been shown that they are regulating the function of cytidine deaminase (CDA), which is a key metabolizer of gemcitabine, thereby contributing to gemcitabine-based chemotherapy resistance [82].

#### 3.4.3. Neutrophils

Neutrophils are recruited into the TME by the interaction of CXCR2 with CXCL1-2 [83]. Like macrophages, neutrophils in the TME can polarize into either the N1 or N2 phenotype, although these phenotypes form a spectrum rather than a discrete distinction. After infiltrating the tumor and converting into either phenotype, neutrophils are termed tumor-associated neutrophils (TANs). At one end of the spectrum, N1 TANs, induced by IFN-α, are considered pro-inflammatory and antitumorigenic as they release reactive oxygen species (ROS), Fas and intracellular adhesion molecule (ICAM)-1. They act as cytotoxic cells against the tumor and hinder immunosuppression within the TME, mainly recruiting and activating CD8^+^ T cells by the secretion of IL-12, TNF-α, CCL3, CCL9 and CXCL10 [84]. At the other end of the spectrum, N2 TANs, induced by TGF-β, promote tumorigenesis by remodeling the extracellular matrix (ECM), inducing angiogenesis, tumor invasion and metastasis through the secretion of various factors, such as arginase (ARG), metalloproteinases (MMPs), and vascular endothelial growth factor (VEGF) [85,86]. Generally, high numbers of neutrophils are correlated with a low number of lymphocytes, which serves as a marker for poor prognosis in PDAC patients [87]. One major feature of mature human neutrophils is the presence of cytoplasmic granules, namely azurophilic, secondary and tertiary granules. While azurophilic granules are important for defense against microbes, secondary and tertiary granules contain proteins that interact and degrade the ECM. As they contain high amounts of MMP-9, they play a major role in shaping the TME of PDAC, mainly by enhancing the invasion of tumor cells, as well as the angiogenesis by inducing VEGF production. Tertiary granules are also responsible for the suppression of CD3-mediated T cell activation and proliferation via the release of ARG-1 [88,89]. Another unique feature of neutrophils is their ability to form neutrophil extracellular traps (NETs), which are composed of DNA fibers released together with proteolytic enzymes to fight against microbes. Recent studies have shown that the formation of NETs not only protects against pathogens, but also contributes to the development of sterile inflammatory diseases such as PDAC [90], and the development of metastasis. The treatment of PDAC mouse models with NET inhibitors, such as DNase I, was able to decrease the number of CAFs that accumulate in the metastatic microenvironment and suppress liver metastasis [90]. Overall, these studies show that neutrophils appear rather tumor-promoting than tumor-suppressing in PDAC and they may play a role in shaping the TME to the advantage of the tumor.

#### 3.4.4. Myeloid-Derived Suppressor Cells

Myeloid-derived suppressor cells (MDSCs) are immature myeloid cells that form a heterogenous population. They are mainly divided into granulocytic or polymorphonuclear (PMN-MDSCs) that are similar to neutrophils, and monocytic (M-MDSCs), which are similar to monocytes [91]. MDSCs are rare in the healthy pancreas, but as PDAC progresses and becomes invasive, their number increases. They are not located in a specific region of the tumor, but are dispersed throughout the invasive tumor [63,92]. Cancer cells contribute to the differentiation of suppressive MDSCs by expressing GM-CSF [93]. In addition, activated PSCs can release the cytokine IL-6, which activates the JAK2/STAT3 signaling cascade, triggering the differentiation of immature myeloid cells into MDSCs [94]. In the TME, MDSCs suppress CD4^+^ and CD8^+^ T cell function through PD-L1 interaction leading to the suppression of T cell activation and tumor tolerance [95]. Moreover, it has been shown that MDSCs stimulate the expansion of Tregs through TGF-β and IL-10 secretion [96]. Interestingly, MDSCs targeted depletion in murine models induced the activation of effective antitumor T cell response in developing tumors and impaired the initiation of Kras^G12D^-driven PDAC tumors [92,97]. In other cancer types, such as lung and breast cancer, MDSCs have been shown to inhibit NK cells through cell–cell mechanisms as well as to promote the polarization of macrophages towards an M2-like phenotype [98,99]. Altogether, these findings suggest that MDSCs play a central role in the inhibition of tumor-specific immune responses and represent an attractive target for the development of new therapeutic treatments.

#### 3.4.5. Natural Killer Cells

NK cells derive from CD34^+^ hematopoietic progenitor cells in the BM and account for 5%–20% of all peripheral blood mononuclear cells. They are characterized by the expression of the neural cell adhesion molecule (NCAM/CD56), the natural cytotoxicity receptor (NCR) NKp46, and the lack of T cell receptor expression [100,101]. NK cells are known to be critical during immune response, especially to defend against viruses and to control tumor growth. To allow self-tolerance, normal healthy cells express MHC class I, which is recognized by and binds to inhibitory receptors on NK cells. However, virus-infected cells or tumor cells are able to downregulate their MHC class I expression to avoid recognition by T cells. This in turn results in less inhibitory signaling to NK cells and the killing of infected or tumor cells. Moreover, viral infection or malignancy induces cellular stress, upregulating ligands for activating receptors on NK cells [102]. However, it was recently suggested that soluble factors or tumor cell-derived extracellular vesicles from the TME can functionally alter NK cells, preventing the recognition and killing of tumor cells [103]. In PDAC, the role of NK cells is only partially understood. The number of NK cells in the peripheral blood circulation of PDAC patients is positively correlated with survival, but their cytotoxicity is reduced compared to those of healthy patients [104]. NK cells from PDAC patients showed a reduced production of granzyme B and perforin, which are components of the cytotoxic granule and key mediators to eliminate cancer cells [105]. PDAC cancer cells also actively suppress NK cell function by expressing several mediators, such as TGF-β, IL-10, IDO, and MMPs, all impairing NK cell recognition and killing [106]. Moreover, the chemokine receptor CXCR2, which is important for NK cell recruitment, is downregulated in NK cells from PDAC patients, leading to impaired tumor infiltration. This explains the low NK cell infiltration (<0.5%) in PDAC.

#### 3.4.6. Adaptive Immune Cells

We will now focus on the different adaptive immune cells and briefly discuss their interactions with other immune, stromal, and cancer cells during the development of PDAC. Adaptive immune cells are as important as innate immune cells in mediating PDAC development. For example, Ino et al. showed that the ratio of Tregs to CD4^+^ T cells (% Tregs) or higher levels of M2 macrophages within the TME are associated with shorter survival, while higher levels of tumor-infiltrating CD4^+^ T and CD8^+^ T cells, or the ratio of M1 macrophages to pan-macrophages (% M1) correlate with longer survival [107]. These results also highlight the importance of the interplay between innate and adaptive immunity during PDAC development.

#### 3.4.7. CD4^+^ T Cells

The CD4^+^ population of lymphocytes is classically divided into four major subpopulations, each with its own distinct features. Helper T (Th) cells are divided into Th1, Th2 and Th17. Th1 cells are promoting cellular type I immunity, including recruitment, priming and the activation of CTLs, M1 macrophages and NK cells, e.g., via the cytokine IFN-γ. A Th1-mediated immune response is required for immunity against intracellular pathogens and tumors. On the other hand, Th2 cells are coordinating the humoral type 2 immunity, characterized by the induction of M2 macrophages against helminths. Furthermore, Th17 cells are required for the defense against extracellular bacteria and fungi. Finally, FoxP3^+^ Treg are characterized by their anti-inflammatory features and induce immune tolerance to avoid an overreaction of the immune system. In pancreatic cancer, the tumor is predominantly infiltrated by Th2 cells compared to Th1 cells, and this trend increases with disease progression [108]. Th2 cells are suggested to have a tumor-promoting activity, as it has been shown that one of the key cytokines they secrete, IL-4, promotes the proliferation of human pancreatic cancer cells [109]. Th2 cells are recruited in an indirect way. First, cancer cells and TAMs produce TNFα and IL1-β, which activate CAFs to produce thymic stromal lymphopoietin (TSLP) [110]. Then, TSLP in turn activates DCs expressing the receptor for TSLP, which finally recruits and induces Th2 cells via the release of CCL17 and CCL22 [111]. The number of Th17 cells is also increased in pancreatic tumors compared to normal adjacent tissue and increasing levels are observed as the disease progresses. In addition, the increased number of Th17 cells in the tumor correlates with a shorter survival and the development of metastasis. By comparing PDAC patients with healthy individuals, it has been observed that systemic levels of IL-17 are increased in PDAC patients and also correlate with cancer severity [112]. Interestingly, IL-17 is also involved in the development of precancerous lesions such as PanIN. The inhibition of IL-17 prevented the formation of PanIN, whereas the induced IL-17 expression accelerated their development [113]. Finally, the number of Tregs in the peripheral blood, as well as in the TME, is increased in PDAC patients [114]. They may play a role in the patient survival as a low number of Tregs correlates with an increased number of infiltrating CTLs and a better prognosis [107]. Interestingly, an increased number of Tregs has also been observed very early in the disease onset, namely during the formation of premalignant lesions, such as IPMNs, and their ratio to CD8^+^ T cells markedly increased during disease progression [115]. The precise function of Tregs in the immune modulation of PDAC is still unknown. However, Jang et al. presented one mechanism whereby Tregs promote PDAC development via the suppression of CD8^+^ T cell-dependent antitumor immunity. They showed that tumor infiltrating Tregs interact for an extended time with tumor-associated CD11c^+^ DCs, reducing their ability to activate T cells. The depletion of Tregs leads to a restored immunogenic tumor-associated CD11c^+^ DC population, resulting in increased CD8^+^ T cell activation, delayed tumor growth and prolonged overall survival [70]. However, the role of Tregs in PDAC may be more complex than expected as it has been recently shown that Treg depletion in distinct mouse models does not relieve immunosuppression, but instead leads to tumor progression. First, as Tregs are a key source of TGF-β, their depletion contributes to the loss of tumor-restraining fibroblasts. Second, it has been observed that the chemokines CCL3, CCL6, and CCL8 are increased upon Treg depletion, leading to an increased myeloid cell recruitment, the restoration of immune suppression, and the promotion of carcinogenesis [116]. These contradictory results show that the role of Tregs in PDAC may not be as straightforward as previously thought, and more studies are needed to understand their precise role in pancreatic cancer. Targeting Tregs as a therapeutic approach may require a combination of multiple treatments.

#### 3.4.8. CD8^+^ T Cells

CD8^+^ T cells are known to be crucial members of the adaptive immune response to defend against intracellular pathogens and cancer cells by producing IFN-γ, TNF and cytotoxic molecules such as perforin and granzymes. During chronic infection or in cancer, CD8^+^ T cells can lose their cytotoxic function and become exhausted CD8^+^ T cells (Tex). Tex are characterized by a progressive loss of effector functions, high and sustained expression of inhibitory receptors, such as programmed cell death 1 (PD1) and cytotoxic T-lymphocyte-associated protein 4 (CTLA-4), distinct transcriptional and epigenetic programs, and metabolic dysregulation. However, as we will discuss later, immune checkpoint blockade (ICB) against PD1 and CTLA-4 failed to improve PDAC patient survival. The levels of Tregs are generally increased in PDAC patients, decreasing the number of CD8^+^ effector cells [115] and the remaining show minimal activation [63], suggesting an exhausted phenotype. Consequently, the number of CD8^+^ T cells that can be rescued by ICB may be too low in PDAC patients to be effective in eliminating tumor cells. Similarly, the frequency of tumor infiltrating CD8^+^ T cells positively correlates with PDAC patient survival [117]. Therefore, understanding the key mechanisms regulating CD8^+^ T cell infiltration will be important to design new immunotherapies that could be used in combination with ICB.

### 3.5. Immunotherapy

Under normal physiological conditions, immune checkpoints are required to regulate and resolve an immune response and maintain self-tolerance, preventing autoimmune diseases. However, in cancer, the upregulation of these inhibitory molecules leads to T cell exhaustion. Immune checkpoint molecules can be expressed by tumor cells to prevent an effective tumor-specific immune response. The best-known checkpoint molecules are CTLA-4 [118] and its ligands B7.1 and B7.2, as well as PD-1 [119] and its ligands PD-L1 and PD-L2 [120]. Blocking CTLA-4 and PD-1 has been shown to reverse T cell exhaustion following chronic presentation of tumor antigens, leading to tumor killing [121,122]. ICB shows remarkable efficacy in many cancers, including lung cancer, renal-cell carcinoma and melanoma [123,124,125,126]. Unfortunately, these positive results could not be translated into PDAC as ICB has minimal impact on the progression of PDAC [123,127].

The main reasons for the failure of ICB in PDAC are the low proportion of tumor infiltrating PD-1^+^ T cells [63,128] and the paucity of neoepitopes [129,130], both of which can predict response to PD-1 blockade in other solid tumors [131,132]. However, in a minor subset of PDAC patients (~1%) with a high epitope burden, PD-1 blockade is effective and has been approved by the FDA [133,134,135]. However, for most PDAC patients, PD-1 or CTLA-4 blockade alone does not appear to be a solution, as both treatments have been tested in phase II trials but failed to show a response [123,127].

As discussed previously, CAFs are part of the immune cell lineage, but their ability to modulate the immune response makes them attractive targets to relieve immunosuppression. In a recent study, Koikawa et al. demonstrated that targeting the proline isomerase Pin1, which they showed to be expressed on CAFs and cancer cells, in combination with anti-PD1 and gemcitabine, induces promising responses in multiple PDAC models. To perform the experiments, they used patient-derived organoids as well as orthotopic allograft models in syngeneic, immunocompetent hosts and treated them with a pharmacologic inhibitor of Pin1 (Pin1i). Consequently, they observed that CAF activation was blocked, the number of immunosuppressive Tregs and myeloid cells were decreased, resulting in an increased number of infiltrating CD8^+^ T cells. In addition, Pin1i seemed to synergize with common chemo- and immunotherapies, such as anti-PD1 and gemcitabine. Indeed, 87.5% of mice treated with the combination of Pin1i, anti-PD1 and gemcitabine showed complete tumor regression and long-term, tumor-free survival. In addition, they showed that high Pin1 expression correlates with a low survival rate, increased Tregs and M2-like macrophages, whereas Pin1-low tumors were characterized by higher cytotoxic CD8^+^ T cells infiltration [136].

Moreover, part of the dysfunctional immune response in PDAC is due to suppressive immune cells, regulated by various cytokines, chemokines and their receptors, providing potential therapeutic targets [97]. The importance of myeloid cells is exemplified by agonistic CD40 therapy, which has been shown to convert macrophages into a more tumoricidal phenotype, resulting in a short-term clinical response [137]. The most clinically advanced target of myeloid cells is CCR2, a chemokine receptor responsible for the recruitment of inhibitory macrophages in the TME which is associated with poor prognosis [43]. In murine models of PDAC, CCR2 inhibition has been shown to improve the response to chemotherapy, inhibit metastasis spread and block monocyte recruitment which increased T cell immune infiltration [138]. The CCR2 inhibitor PF-04136309 underwent a phase I study in PDAC patients treated with FOLFIRINOX and resulted in an objective response in almost half of the patients [139]. Additional cytokine and chemokine receptors have recently drawn attention, such as CSF-1R, the receptor for M-CSF which acts as a key regulator of MDSCs and TAMs in PDAC, as well as CXCR2, which is responsible for neutrophile and MDSC migration. The preclinical analysis of CSF-1R in combination with checkpoint blockade showed promising results [140]. Moreover, the combination of CSF-1R inhibition with CD40 agonism was able to induce potent T cell-mediated tumor killing in a melanoma model [141]. The inhibition of CXCR2 alone resulted in enhanced T cell infiltration, and when combined with checkpoint blockade or CSF-1R inhibition, tumor responses were enhanced [142,143]. Since the cytotoxic activity of NK cells is impaired in PDAC, they may represent potential target cells for the development of new immunotherapies. With this idea in mind, two clinical trials have demonstrated that combining allogeneic NK cell immunotherapy with percutaneous irreversible electroporation increases the DFS and OS of stage III PDAC patients and extends the OS of stage IV PDAC patients [144]. In mouse models, it has been shown that gemcitabine efficacy was dependent on an increase in NK cell tumor infiltration, together with a decrease in MDSCs [145]. Therefore, NK cell-targeted immunotherapy seems promising but may require a combination of therapies to overcome the immunosuppression and restore their full activity. Finally, the immunosuppressive enzyme produced by DCs and MDSCs, IDO1, contributes to T cell dysfunction. Hence, a combination of IDO1 inhibition and hyaluronidase resulted in improved T cell infiltration and remission in preclinical models [146]. Alone or in combination, these molecules promise to improve the dismal prognosis of PDAC, and various combinations are under investigation.

### 3.6. Cancer Cell-Based Vaccines

As cancer cells can escape the immune response to some extent, one strategy is to present tumor antigens to the immune system in the form of a tumor-based vaccine to induce a specific response against the tumor. Therapeutic vaccines hold the potential to elicit a strong tumor-specific immune response; however, so far, they have failed to provide long-term benefits for PDAC patients. These vaccines can consist of whole-tumor cells, peptides, proteins, or recombinant constructs. The goal is to prime and activate tumor-specific T cells that target and eliminate tumor cells. In phase I trials, all the different vaccines promoted tumor-specific T cell responses but showed no long-term survival benefit [147,148,149]. Overall, these results have diminished hope for therapeutic vaccines as a treatment for PDAC. Despite the disappointing results of these trials, some interesting observations have been made. Namely, these trials have demonstrated that vaccines can break tolerance and generate T cell immunity to tumor-associated self-antigens without obvious short-term side effects. The GVAX vaccine, which consists of irradiated whole tumor cells which have been genetically modified to secrete GM-CSF, was even able to induce the formation of tertiary lymphoid structures and the infiltration of T cells in numerous patients [150]. The reason for the lack of improvement in survival remains unclear. One possible explanation is that while CD8^+^ T cells are capable of infiltrating tumors, they can also promote the recruitment of Tregs. In fact, there is evidence that dysfunctional T cells can be rescued by the depletion of Tregs, thus enabling therapeutic vaccination [151]. Moreover, in human GVAX-induced T cells, checkpoint molecules such as PD-1 are upregulated [150], suggesting that GVAX in combination with checkpoint blockade could functionally rescue CD8^+^ T cells. Combinations of therapeutic vaccines with the checkpoint blockade are currently being tested [152].

## 4. Conclusions and Outlook

As discussed in this review, some single treatments are effective to some extent, such as FOLFIRINOX, but most of them have failed to meet expectations, although they have been able to elicit a response to the tumor. One possible explanation is that, so far, no major driver of PDAC progression has been found, but rather an extensive number of aspects contributing to tumorigenesis. As discussed, immunosuppression in PDAC is not induced by a single cell type or component, but by a combination of cells such as Tregs, MDSCs or CAFs, secreting multiple tumor-promoting mediators such as IL-10 or MMP9. It may then seem attractive to direct our research towards combination therapies. A potential therapeutic approach is to combine ICB and chemotherapy with small inhibitory molecules blocking immunosuppression, thereby inducing cancer cell death, while promoting a potent and sustained immune response against the tumor. The concept of combination therapy is exemplified by the previously mentioned phase I clinical trial with the combination of FOLFIRINOX and an inhibitor of CCR2 which resulted in local tumor control in 97% of the patients with BRPC and locally advanced PDAC. In a preclinical model of PDAC, Winograd et al. combined agonist anti-CD40 mAb with ICB. This combination induced T-cell immunity, the regression of subcutaneous tumors, an improved survival, and conferred protection against tumor rechallenge, showing some degree of immune memory. In mice with spontaneous tumors, this combination nearly doubled their survival, although they were not cured.

As tumor heterogeneity among patients is very high compared to other tumors, personalized therapy for individual patients or groups of patients based on the genotypic and phenotypic heterogeneity of PDAC may contribute to appropriate and effective treatment strategies. As previously discussed, it has been shown that PDAC can be divided into two subtypes based on RNA transcriptional analyses, namely basal-like and classical [11]. Clinical trials have already shown the beneficial results of treating patients based on this stratification. In the COMPASS trial, advanced PDAC patients were classified as basal-like or classical based on whole genome sequencing (WGS) or RNAseq analyses. Patients of the classical subtype showed a better response to first-line chemotherapy (FOLFIRINOX 8.5 months) [153]. The genomic instability and the relationship with DNA maintenance genes, such as *BRCA1/2*, may be another way to stratify the tumors as approximately 4%–7% of PDAC patients have a germline *BRCA* mutation [154,155]. BRCA genes code for proteins that are required for the proper homologous recombination repair (HRR) of DNA double-strand breaks. Cells that are deficient for HRR, such as those presenting a mutation in *BRCA* genes, *PALB2* (partner and localizer of BRCA2), *ATM*, and *CHEK2* (checkpoint kinase 2) are sensitive to platinum-based and poly (ADP-ribose) polymerase (PARP) inhibitors. PARP inhibitors function by binding on DNA at sites of single-strand breaks, preventing the repair and leading to the generation of double-strand breaks in replicating cells. These double-strand breaks cannot be repaired in HRR-deficient cells, causing the accumulation of DNA damage and cell death [156]. A successful clinical trial with germline BRCA-mutated metastatic PDAC patients has led to the approval of the targeted agent Olaparib for these patients [157]. Another promising method to address tumor heterogeneity and predict treatment response in PDAC is precision medicine based on organoid cultures derived from patient tumor samples. Indeed, patient-derived organoids showed strong concordance for histopathologic features and protein markers, such as claudin 4 and CA19-9, as well as patient-specific genomic and transcriptomic profiles. Patient-specific responses to FOLFIRINOX or gemcitabine in xenografts were recapitulated *in vitro* with organoids [158]. Therefore, patient-derived organoids are useful tools for predicting response to drug combinations and dosage [159].

Overall, major steps to improve our understanding of PDAC have been made thanks to clinical and basic research, and at the same time, patient care has improved. However, some important mechanisms in PDAC remain unknown, preventing the development of effective therapies. Nevertheless, recent success in the field of immunotherapies and in the understanding of the crosstalk between tumor, immune, and stromal cells may soon lead to major discoveries in this field and give hope to patients diagnosed with this devastating disease.

## Figures and Tables

**Table 1:** Overview of pancreatic cancer staging.

AJCC Staging	I–II	II–III	II–III	IV
Clinical stage	Resectable	Borderline resectable	Locally advanced	Metastatic
Vascular involvement	No or <180° contact	<180° contact	>180° contact	N/A
Prevalence at diagnosis (~%) ^a^	10–15	30–35	30–35	50
Treatment intent	Curative	Curative	Palliative	Palliative
5-year survival rate (~%) ^a^	35–45	10–15	10–15	<5

^a^ Cancer Statistics, 2021; CA CANCER J CLIN 2021;71:7–33; doi: 10.3322/caac.21654.

**Table 2:** Summary of selected clinical trials.

Trial	Therapy Type	Treatment Groups	Number of Patients	Median Survival in Months	Year of Publication
ESPAC-1	Adjuvant	5-FU vs. resection only	188	19.7 vs. 14	2001
CONKO-001	Adjuvant	Gemcitabine vs. resection only	368	13.4 vs. 6.7	2007
ESPAC-3	Adjuvant	5-FU + folinic acid vs. gemcitabine	1088	23 vs. 23.6	2010
PRODIGE	First line for stage IV	FOLFIRINOX vs. gemcitabine	342	11.1 vs. 6.8	2011
PRODIGE-24	Adjuvant	FOLFIRINOX vs. Gemcitabine	493	54.4 vs. 35	2017

## References

[1] Strobel O., Neoptolemos J., Jäger D., Büchler M.W. (2019). Optimizing the outcomes of pancreatic cancer surgery. Nat. Rev. Clin. Oncol..

[2] Wolfgang C.L., Herman J.M., Laheru D.A., Klein A.P., Erdek M.A., Fishman E.K., Hruban R.H. (2013). Recent progress in pancreatic cancer. CA Cancer J. Clin..

[3] Ryan D.P., Hong T.S., Bardeesy N. (2014). Pancreatic Adenocarcinoma. N. Engl. J. Med..

[4] Yadav D., Lowenfels A.B. (2013). The epidemiology of pancreatitis and pancreatic cancer. Gastroenterology.

[5] Genkinger J.M., Spiegelman D., Anderson K.E., Bergkvist L., Bernstein L., van den Brandt P.A., English D.R., Freudenheim J.L., Fuchs C.S., Giles G.G. (2009). Alcohol Intake and Pancreatic Cancer Risk: A Pooled Analysis of Fourteen Cohort Studies. Cancer Epidemiol. Prev. Biomark..

[6] Li D., Xie K., Wolff R., Abbruzzese J.L. (2004). Pancreatic cancer. Lancet.

[7] Henley S.J., Ward E.M., Scott S., Ma J., Anderson R.N., Firth A.U., Thomas C.C., Islami F., Weir H.K., Lewis D.R. (2020). Annual report to the nation on the status of cancer, part I: National cancer statistics. Cancer.

[8] Hruban R.H., Maitra A., Goggins M. (2020). Pancreatic Intraepithelial Neoplasia. Definitions.

[9] Takaori K. (2007). Current understanding of precursors to pancreatic cancer. J. Hepatobiliary Pancreat. Surg..

[10] Biankin A.V., Maitra A. (2015). Subtyping Pancreatic Cancer. Cancer Cell.

[11] Moffitt R.A., Marayati R., Flate E.L., Volmar K.E., Loeza S.G.H., Hoadley K.A., Rashid N.U., Williams L.A., Eaton S.C., Chung A.H. (2015). Virtual microdissection identifies distinct tumor- and stroma-specific subtypes of pancreatic ductal adenocarcinoma. Nat. Genet..

[12] Edge S.B., Byrd D.R., Carducci M.A., Compton C.C., Fritz A.G., Greene F.L. (2010). AJCC Cancer Staging Manual.

[13] Bilimoria K.Y., Bentrem D.J., Ko C.Y., Ritchey J., Stewart A.K., Winchester D.P., Talamonti M.S. (2007). Validation of the 6th edition AJCC pancreatic cancer staging system: Report from the National Cancer Database. Cancer.

[14] Lopez N.E., Prendergast C., Lowy A.M. (2014). Borderline resectable pancreatic cancer: Definitions and management. World J. Gastroenterol..

[15] National Comprehensive Cancer Network (2021). Practice Guidelines in Oncology for Pancreatic Adenocarcinoma.

[16] Esposito I., Kleeff J.J., Bergmann F., Reiser C., Herpel E., Friess H., Schirmacher P., Büchlerbüchler M.W. (2008). Most Pancreatic Cancer Resections are R1 Resections. Ann. Surg. Oncol..

[17] Ferrone C.R., Marchegiani G., Hong T.S., Ryan D.P., Deshpande V., McDonnell E.I., Sabbatino F., Santos D.D., Allen J.N., Blaszkowsky L.S. (2015). Radiological and surgical implications of neoadjuvant treatment with FOLFIRINOX for locally advanced and borderline resectable pancreatic cancer. Ann. Surg..

[18] Alliance for Clinical Trials in Oncology (2020). Testing the Use of the Usual Chemotherapy before and after Surgery for Removable Pancreatic Cancer.

[19] Fatima J. (2010). Pancreatoduodenectomy for Ductal Adenocarcinoma. Arch. Surg..

[20] Delpero J.R., Boher J.M., Sauvanet A., Le Treut Y.P., Sa-Cunha A., Mabrut J.Y., Chiche L., Turrini O., Bachellier P., Paye F. (2015). Pancreatic Adenocarcinoma with Venous Involvement: Is Up-Front Synchronous Portal-Superior Mesenteric Vein Resection Still Justified? A Survey of the Association Française de Chirurgie. Ann. Surg. Oncol..

[21] Petrelli F., Coinu A., Borgonovo K., Cabiddu M., Ghilardi M., Lonati V., Aitini E., Barni S. (2015). FOLFIRINOX-based neoadjuvant therapy in borderline resectable or unresectable pancreatic cancer: A meta-analytical review of published studies. Pancreas.

[22] Versteijne E., Vogel J.A., Besselink M.G., Busch O.R.C., Wilmink J.W., Daams J.G., van Eijck C.H.J., Groot Koerkamp B., Rasch C.R.N., van Tienhoven G. (2018). Meta-analysis comparing upfront surgery with neoadjuvant treatment in patients with resectable or borderline resectable pancreatic cancer. Br. J. Surg..

[23] Neoptolemos J.P., Stocken D.D., Friess H., Bassi C., Dunn J.A., Hickey H., Beger H., Fernandez-Cruz L., Dervenis C., Lacaine F. (2004). A randomized trial of chemoradiotherapy and chemotherapy after resection of pancreatic cancer. N. Engl. J. Med..

[24] Oettle H., Post S., Neuhaus P. (2007). Vs Observation in Patients Undergoing Curative-Intent Resection of Pancreatic Cancer. JAMA.

[25] Oettle H., Neuhaus P., Hochhaus A., Hartmann J.T., Gellert K., Ridwelski K., Niedergethmann M., Zülke C., Fahlke J., Arning M.B. (2013). Adjuvant chemotherapy with gemcitabine and long-term outcomes among patients with resected pancreatic cancer: The CONKO-001 randomized trial. JAMA J. Am. Med. Assoc..

[26] Neoptolemos J.P., Stocken D.D., Bassi C. (2010). Adjuvant Chemotherapy with Fluorouracil Plus Folinic Acid vs. Gemcitabine Following Pancreatic Cancer Resection. JAMA J. Am. Med. Assoc..

[27] Conroy T., Hammel P., Turpin A., Belletier C., Wei A., Mitry E., Lopez A., Francois E., Artru P., Biagi J. (2021). LBA57 Unicancer PRODIGE 24/CCTG PA6 trial: Updated results of a multicenter international randomized phase III trial of adjuvant mFOLFIRINOX (mFFX) versus gemcitabine (gem) in patients (pts) with resected pancreatic ductal adenocarcinomas (PDAC). Ann. Oncol..

[28] Tempero M.A., Malafa M.P., Al-Hawary M., Behrman S.W., Benson A.B., Cardin D.B., Chiorean E.G., Chung V., Czito B., Del Chiaro M. (2021). Pancreatic Adenocarcinoma, Version 2.2021, NCCN Clinical Practice Guidelines in Oncology. J. Natl. Compr. Cancer Netw..

[29] Loehrer Sr P.J., Feng Y., Cardenes H., Wagner L., Brell J.M., Cella D., Flynn P., Ramanathan R.K., Crane C.H., Alberts S.R. (2011). Gemcitabine Alone Versus Gemcitabine Plus Radiotherapy in Patients with Locally Advanced Pancreatic Cancer: An Eastern Cooperative Oncology Group Trial. J. Clin. Oncol..

[30] Von Hoff D.D., Ervin T., Arena F.P., Chiorean E.G., Infante J., Moore M., Seay T., Tjulandin S.A., Wee Ma W., Saleh N. (2013). Increased Survival in Pancreatic Cancer with nab-Paclitaxel plus Gemcitabine. N. Engl. J. Med..

[31] Goldstein D., El-Maraghi R.H., Hammel P., Heinemann V., Kunzmann V., Sastre J., Scheithauer W., Siena S., Tabernero J., Teixeira L. (2015). nab-Paclitaxel Plus Gemcitabine for Metastatic Pancreatic Cancer: Long-Term Survival From a Phase III Trial. JNCI J. Natl. Cancer Inst..

[32] Cho I.R., Kang H., Jo J.H., Lee H.S., Chung M.J., Park J.Y., Park S.W., Song S.Y., An C., Park M.-S. (2020). FOLFIRINOX vs gemcitabine/nab-paclitaxel for treatment of metastatic pancreatic cancer: Single-center cohort study. World J. Gastrointest. Oncol..

[33] Burris H.A., Moore M.J., Andersen J., Green M.R., Rothenberg M.L., Modiano M.R., Cripps M.C., Portenoy R.K., Storniolo A.M., Tarassoff P. (1997). Improvements in survival and clinical benefit with gemcitabine as first-line therapy for patients with advanced pancreas cancer: A randomized trial. J. Clin. Oncol..

[34] Moore M.J., Goldstein D., Hamm J., Figer A., Hecht J.R., Gallinger S., Au H.J., Murawa P., Walde D., Wolff R.A. (2007). Erlotinib plus gemcitabine compared with gemcitabine alone in patients with advanced pancreatic cancer: A phase III trial of the National Cancer Institute of Canada Clinical Trials Group. J. Clin. Oncol..

[35] Conroy T., Desseigne F., Ychou M., Bouché O., Guimbaud R., Bécouarn Y., Adenis A., Raoul J.-L., Gourgou-Bourgade S., de la Fouchardière C. (2011). FOLFIRINOX versus Gemcitabine for Metastatic Pancreatic Cancer. N. Engl. J. Med..

[36] Singhal M.K., Kapoor A., Bagri P.K., Narayan S., Singh D., Nirban R.K., Singh G., Maharia S., Kumari P., Jakhar S.L. (2014). A Phase III Trial Comparing Folfirinox Versus Gemcitabine for Metastatic Pancreatic Cancer. Ann. Oncol..

[37] Cunningham D., Chau I., Stocken D.D., Valle J.W., Smith D., Steward W., Harper P.G., Dunn J., Tudur-Smith C., West J. (2009). Phase III randomized comparison of gemcitabine versus gemcitabine plus capecitabine in patients with advanced pancreatic cancer. J. Clin. Oncol..

[38] Li Q., Yan H., Liu W., Zhen H., Yang Y., Cao B. (2014). Efficacy and Safety of Gemcitabine-Fluorouracil Combination Therapy in the Management of Advanced Pancreatic Cancer: A Meta-Analysis of Randomized Controlled Trials. PLoS ONE.

[39] Heinemann V., Boeck S., Hinke A., Labianca R., Louvet C. (2008). Meta-analysis of randomized trials: Evaluation of benefit from gemcitabine-based combination chemotherapy applied in advanced pancreatic cancer. BMC Cancer.

[40] Shelemey P.T., Amaro C.P., Ng D., Falck V., Tam V.C. (2021). Metastatic pancreatic cancer with complete response to FOLFIRINOX treatment. BMJ Case Rep..

[41] Ryschich E., Nötzel T., Hinz U., Autschbach F., Ferguson J., Simon I., Weitz J., Fröhlich B., Klar E., Büchler M.W. (2005). Control of T-Cell-Mediated Immune Response by HLA Class I in Human Pancreatic Carcinoma. Clin. Cancer Res..

[42] Pandha H., Rigg A., John J., Lemoine N. (2007). Loss of expression of antigen-presenting molecules in human pancreatic cancer and pancreatic cancer cell lines. Clin. Exp. Immunol..

[43] Sanford D.E., Belt B.A., Panni R.Z., Mayer A., Deshpande A.D., Carpenter D., Mitchem J.B., Plambeck-Suess S.M., Worley L.A., Goetz B.D. (2013). Inflammatory Monocyte Mobilization Decreases Patient Survival in Pancreatic Cancer: A Role for Targeting the CCL2/CCR2 Axis. Clin. Cancer Res..

[44] Tan M.C.B., Goedegebuure P.S., Belt B.A., Flaherty B., Sankpal N., Gillanders W.E., Eberlein T.J., Hsieh C.-S., Linehan D.C. (2009). Disruption of CCR5-Dependent Homing of Regulatory T Cells Inhibits Tumor Growth in a Murine Model of Pancreatic Cancer. J. Immunol..

[45] Basso D., Fogar P., Falconi M., Fadi E., Sperti C., Frasson C., Greco E., Tamburrino D., Teolato S., Moz S. (2013). Pancreatic Tumors and Immature Immunosuppressive Myeloid Cells in Blood and Spleen: Role of Inhibitory Co-Stimulatory Molecules PDL1 and CTLA4. An In Vivo and In Vitro Study. PLoS ONE.

[46] Uyttenhove C., Pilotte L., Théate I., Stroobant V., Colau D., Parmentier N., Boon T., Van den Eynde B.J. (2003). Evidence for a tumoral immune resistance mechanism based on tryptophan degradation by indoleamine 2,3-dioxygenase. Nat. Med..

[47] Witkiewicz A., Williams T.K., Cozzitorto J., Durkan B., Showalter S.L., Yeo C.J., Brody J.R. (2008). Expression of Indoleamine 2,3-Dioxygenase in Metastatic Pancreatic Ductal Adenocarcinoma Recruits Regulatory T Cells to Avoid Immune Detection. J. Am. Coll. Surg..

[48] Fallarino F., Grohmann U., You S., McGrath B.C., Cavener D.R., Vacca C., Orabona C., Bianchi R., Belladonna M.L., Volpi C. (2006). The Combined Effects of Tryptophan Starvation and Tryptophan Catabolites Down-Regulate T Cell Receptor ζ-Chain and Induce a Regulatory Phenotype in Naive T Cells. J. Immunol..

[49] Mahadevan D., Von Hoff D.D. (2007). Tumor-stroma interactions in pancreatic ductal adenocarcinoma. Mol. Cancer Ther..

[50] Masamune A., Shimosegawa T. (2009). Signal transduction in pancreatic stellate cells. J. Gastroenterol..

[51] Neesse A., Algül H., Tuveson D.A., Gress T.M. (2015). Stromal biology and therapy in pancreatic cancer: A changing paradigm. Gut.

[52] Feig C., Gopinathan A., Neesse A., Chan D.S., Cook N., Tuveson D.A. (2012). The pancreas cancer microenvironment. Clin. Cancer Res..

[53] Hwang R.F., Moore T., Arumugam T., Ramachandran V., Amos K.D., Rivera A., Ji B., Evans D.B., Logsdon C.D. (2008). Cancer-associated stromal fibroblasts promote pancreatic tumor progression. Cancer Res..

[54] Mukherjee P., Basu G.D., Tinder T.L., Subramani D.B., Bradley J.M., Arefayene M., Skaar T., De Petris G. (2009). Progression of Pancreatic Adenocarcinoma Is Significantly Impeded with a Combination of Vaccine and COX-2 Inhibition. J. Immunol..

[55] Infante J.R., Matsubayashi H., Sato N., Tonascia J., Klein A.P., Riall T.A., Yeo C., Iacobuzio-Donahue C., Goggins M. (2007). Peritumoral fibroblast SPARC expression and patient outcome with resectable pancreatic adenocarcinoma. J. Clin. Oncol..

[56] Özdemir B.C., Pentcheva-Hoang T., Carstens J.L., Zheng X., Wu C.C., Simpson T.R., Laklai H., Sugimoto H., Kahlert C., Novitskiy S.V. (2014). Depletion of carcinoma-associated fibroblasts and fibrosis induces immunosuppression and accelerates pancreas cancer with reduced survival. Cancer Cell.

[57] Öhlund D., Handly-Santana A., Biffi G., Elyada E., Almeida A.S., Ponz-Sarvise M., Corbo V., Oni T.E., Hearn S.A., Lee E.J. (2017). Distinct populations of inflammatory fibroblasts and myofibroblasts in pancreatic cancer. J. Exp. Med..

[58] Biffi G., Oni T.E., Spielman B., Hao Y., Elyada E., Park Y., Preall J., Tuveson D.A. (2019). IL1-Induced JAK/STAT Signaling Is Antagonized by TGFβ to Shape CAF Heterogeneity in Pancreatic Ductal Adenocarcinoma. Cancer Discov..

[59] Rhim A.D., Oberstein P.E., Thomas D.H., Mirek E.T., Palermo C.F., Sastra S.A., Dekleva E.N., Saunders T., Becerra C.P., Tattersall I.W. (2014). Stromal elements act to restrain, rather than support, pancreatic ductal adenocarcinoma. Cancer Cell.

[60] Hingorani S.R., Bullock A.J., Seery T.E., Zheng L., Sigal D., Ritch P.S., Braiteh F.S., Zalupski M., Bahary N., Harris W.P. (2017). Randomized phase II study of PEGPH20 plus nab-paclitaxel/gemcitabine (PAG) vs AG in patients (Pts) with untreated, metastatic pancreatic ductal adenocarcinoma (mPDA). J. Clin. Oncol..

[61] Tempero M.A., Van Cutsem E., Sigal D., Oh D.-Y., Fazio N., Macarulla T., Hitre E., Hammel P., Hendifar A.E., Bates S.E. (2020). HALO 109-301: A randomized, double-blind, placebo-controlled, phase 3 study of pegvorhyaluronidase alfa (PEGPH20) + nab-paclitaxel/gemcitabine (AG) in patients (pts) with previously untreated hyaluronan (HA)-high metastatic pancreatic ductal adenocarcinom. J. Clin. Oncol..

[62] Jiang H., Hegde S., Knolhoff B.L., Zhu Y., Herndon J.M., Meyer M.A., Nywening T.M., Hawkins W.G., Shapiro I.M., Weaver D.T. (2016). Targeting focal adhesion kinase renders pancreatic cancers responsive to checkpoint immunotherapy. Nat. Med..

[63] Clark C.E., Hingorani S.R., Mick R., Combs C., Tuveson D.A., Vonderheide R.H. (2007). Dynamics of the immune reaction to pancreatic cancer from inception to invasion. Cancer Res..

[64] Padoan A., Plebani M., Basso D. (2019). Inflammation and Pancreatic Cancer: Focus on Metabolism, Cytokines, and Immunity. Int. J. Mol. Sci..

[65] Tanţău A., Leucuţa D.-C., Tanţău M., Boţan E., Zaharie R., Mândruţiu A., Tomuleasa I.-C. (2020). Inflammation, Tumoral Markers and Interleukin-17, -10, and -6 Profiles in Pancreatic Adenocarcinoma and Chronic Pancreatitis. Dig. Dis. Sci..

[66] Yao W., Maitra A., Ying H. (2020). Recent insights into the biology of pancreatic cancer. EBioMedicine.

[67] Saka D., Gökalp M., Piyade B., Cevik N.C., Arik Sever E., Unutmaz D., Ceyhan G.O., Demir I.E., Asimgil H. (2020). Mechanisms of T-Cell Exhaustion in Pancreatic Cancer. Cancers.

[68] Roshani R., McCarthy F., Hagemann T. (2014). Inflammatory cytokines in human pancreatic cancer. Cancer Lett..

[69] Lei X., Lei Y., Li J.-K., Du W.-X., Li R.-G., Yang J., Li J., Li F., Tan H.-B. (2020). Immune cells within the tumor microenvironment: Biological functions and roles in cancer immunotherapy. Cancer Lett..

[70] Jang J.E., Hajdu C.H., Liot C., Miller G., Dustin M.L., Bar-Sagi D. (2017). Crosstalk between Regulatory T Cells and Tumor-Associated Dendritic Cells Negates Anti-tumor Immunity in Pancreatic Cancer. Cell Rep..

[71] Bellone G., Carbone A., Smirne C., Scirelli T., Buffolino A., Novarino A., Stacchini A., Bertetto O., Palestro G., Sorio C. (2006). Cooperative Induction of a Tolerogenic Dendritic Cell Phenotype by Cytokines Secreted by Pancreatic Carcinoma Cells. J. Immunol..

[72] Bharadwaj U., Li M., Zhang R., Chen C., Yao Q. (2007). Elevated interleukin-6 and G-CSF in human pancreatic cancer cell conditioned medium suppress dendritic cell differentiation and activation. Cancer Res..

[73] Zhou J., Tang Z., Gao S., Li C., Feng Y., Zhou X. (2020). Tumor-Associated Macrophages: Recent Insights and Therapies. Front. Oncol..

[74] Gordon S., Taylor P.R. (2005). Monocyte and macrophage heterogeneity. Nat. Rev. Immunol..

[75] Santoni M., Bracarda S., Nabissi M., Massari F., Conti A., Bria E., Tortora G., Santoni G., Cascinu S. (2014). CXC and CC Chemokines as Angiogenic Modulators in Nonhaematological Tumors. Biomed Res. Int..

[76] Zhu Y., Herndon J.M., Sojka D.K., Kim K.-W., Knolhoff B.L., Zuo C., Cullinan D.R., Luo J., Bearden A.R., Lavine K.J. (2017). Tissue-Resident Macrophages in Pancreatic Ductal Adenocarcinoma Originate from Embryonic Hematopoiesis and Promote Tumor Progression. Immunity.

[77] Biswas S.K., Mantovani A. (2010). Macrophage plasticity and interaction with lymphocyte subsets: Cancer as a paradigm. Nat. Immunol..

[78] Penny H.L., Sieow J.L., Adriani G., Yeap W.H., See Chi Ee P., San Luis B., Lee B., Lee T., Mak S.Y., Ho Y.S. (2016). Warburg metabolism in tumor-conditioned macrophages promotes metastasis in human pancreatic ductal adenocarcinoma. Oncoimmunology.

[79] Kurahara H., Shinchi H., Mataki Y., Maemura K., Noma H., Kubo F., Sakoda M., Ueno S., Natsugoe S., Takao S. (2011). Significance of M2-polarized tumor-associated macrophage in pancreatic cancer. J. Surg. Res..

[80] Zeng L., Guo Y., Liang J., Chen S., Peng P., Zhang Q., Su H., Chen Y., Huang K. (2014). Perineural invasion and TAMs in pancreatic ductal adenocarcinomas: Review of the original pathology reports using immunohistochemical enhancement and relationships with clinicopathological features. J. Cancer.

[81] Kurahara H., Takao S., Maemura K., Mataki Y., Kuwahata T., Maeda K., Sakoda M., Iino S., Ishigami S., Ueno S. (2013). M2-Polarized tumor-associated macrophage infiltration of regional lymph nodes is associated with nodal lymphangiogenesis and occult nodal involvement in pn0 pancreatic cancer. Pancreas.

[82] Weizman N., Krelin Y., Shabtay-Orbach A., Amit M., Binenbaum Y., Wong R.J., Gil Z. (2014). Macrophages mediate gemcitabine resistance of pancreatic adenocarcinoma by upregulating cytidine deaminase. Oncogene.

[83] Nywening T.M., Belt B.A., Cullinan D.R., Panni R.Z., Han B.J., Sanford D.E., Jacobs R.C., Ye J., Patel A.A., Gillanders W.E. (2018). Targeting both tumour-associated CXCR2^+^ neutrophils and CCR2^+^ macrophages disrupts myeloid recruitment and improves chemotherapeutic responses in pancreatic ductal adenocarcinoma. Gut.

[84] Mollinedo F. (2019). Neutrophil Degranulation, Plasticity, and Cancer Metastasis. Trends Immunol..

[85] Mizuno R., Kawada K., Itatani Y., Ogawa R., Kiyasu Y., Sakai Y. (2019). The Role of Tumor-Associated Neutrophils in Colorectal Cancer. Int. J. Mol. Sci..

[86] Masucci M.T., Minopoli M., Carriero M.V. (2019). Tumor Associated Neutrophils. Their Role in Tumorigenesis, Metastasis, Prognosis and Therapy. Front. Oncol..

[87] Felix K., Gaida M.M. (2016). Neutrophil-Derived Proteases in the Microenvironment of Pancreatic Cancer-Active Players in Tumor Progression. Int. J. Biol. Sci..

[88] Wang Y., Fang T., Huang L., Wang H., Zhang L., Wang Z., Cui Y. (2018). Neutrophils infiltrating pancreatic ductal adenocarcinoma indicate higher malignancy and worse prognosis. Biochem. Biophys. Res. Commun..

[89] Ardi V.C., Kupriyanova T.A., Deryugina E.I., Quigley J.P. (2007). Human neutrophils uniquely release TIMP-free MMP-9 to provide a potent catalytic stimulator of angiogenesis. Proc. Natl. Acad. Sci. USA.

[90] Takesue S., Ohuchida K., Shinkawa T., Otsubo Y., Matsumoto S., Sagara A., Yonenaga A., Ando Y., Kibe S., Nakayama H. (2020). Neutrophil extracellular traps promote liver micrometastasis in pancreatic ductal adenocarcinoma via the activation of cancer-associated fibroblasts. Int. J. Oncol..

[91] Thyagarajan A., Alshehri M.S.A., Miller K.L.R., Sherwin C.M., Travers J.B., Sahu R.P. (2019). Myeloid-Derived Suppressor Cells and Pancreatic Cancer: Implications in Novel Therapeutic Approaches. Cancers.

[92] Stromnes I.M., Brockenbrough J.S., Izeradjene K., Carlson M.A., Cuevas C., Simmons R.M., Greenberg P.D., Hingorani S.R. (2014). Targeted depletion of an MDSC subset unmasks pancreatic ductal adenocarcinoma to adaptive immunity. Gut.

[93] Bayne L.J., Beatty G.L., Jhala N., Clark C.E., Rhim A.D., Stanger B.Z., Vonderheide R.H. (2012). Tumor-Derived Granulocyte-Macrophage Colony-Stimulating Factor Regulates Myeloid Inflammation and T Cell Immunity in Pancreatic Cancer. Cancer Cell.

[94] Mace T.A., Bloomston M., Lesinski G.B. (2013). Pancreatic cancer-associated stellate cells: A viable target for reducing immunosuppression in the tumor microenvironment. Oncoimmunology.

[95] Pinton L., Solito S., Damuzzo V., Francescato S., Pozzuoli A., Berizzi A., Mocellin S., Rossi C.R., Bronte V., Mandruzzato S. (2016). Activated T cells sustain myeloid-derived suppressor cellmediated immune suppression. Oncotarget.

[96] Huang B., Pan P.Y., Li Q., Sato A.I., Levy D.E., Bromberg J., Divino C.M., Chen S.H. (2006). Gr-1^+^CD115^+^ immature myeloid suppressor cells mediate the development of tumor-induced T regulatory cells and T-cell anergy in tumor-bearing host. Cancer Res..

[97] Zhang Y., Velez-Delgado A., Mathew E., Li D., Mendez F.M., Flannagan K., Rhim A.D., Simeone D.M., Beatty G.L., Di Magliano M.P. (2017). Myeloid cells are required for PD-1/PD-L1 checkpoint activation and the establishment of an immunosuppressive environment in pancreatic cancer. Gut.

[98] Liu C., Yu S., Kappes J., Wang J., Grizzle W.E., Zinn K.R., Zhang H.G. (2007). Expansion of spleen myeloid suppressor cells represses NK cell cytotoxicity in tumor-bearing host. Blood.

[99] Sinha P., Clements V.K., Bunt S.K., Albelda S.M., Ostrand-Rosenberg S. (2007). Cross-Talk between Myeloid-Derived Suppressor Cells and Macrophages Subverts Tumor Immunity toward a Type 2 Response. J. Immunol..

[100] Chiossone L., Dumas P.-Y., Vienne M., Vivier E. (2018). Natural killer cells and other innate lymphoid cells in cancer. Nat. Rev. Immunol..

[101] Lanier L.L., Testi R., Bindl J., Phillips J.H. (1989). Identity of Leu-19 (CD56) leukocyte differentiation antigen and neural cell adhesion molecule. J. Exp. Med..

[102] Lanier L.L. (2008). Up on the tightrope: Natural killer cell activation and inhibition. Nat. Immunol..

[103] Liu S., Dhar P., Wu J.D. (2019). NK Cell Plasticity in Cancer. J. Clin. Med..

[104] Davis M., Conlon K., Bohac G.C., Barcenas J., Leslie W., Watkins L., Lamzabi I., Deng Y., Li Y., Plate J.M.D. (2012). Effect of Pemetrexed on Innate Immune Killer Cells and Adaptive Immune T Cells in Subjects with Adenocarcinoma of the Pancreas. J. Immunother..

[105] Funa K., Nilsson B., Jacobsson G., Alm G.V. (1984). Decreased natural killer cell activity and interferon production by leucocytes in patients with adenocarcinoma of the pancreas. Br. J. Cancer.

[106] Peng Y.P., Zhang J.J., Liang W.B., Tu M., Lu Z.P., Wei J.S., Jiang K.R., Gao W.T., Wu J.L., Xu Z.K. (2014). Elevation of MMP-9 and IDO induced by pancreatic cancer cells mediates natural killer cell dysfunction. BMC Cancer.

[107] Ino Y., Yamazaki-Itoh R., Shimada K., Iwasaki M., Kosuge T., Kanai Y., Hiraoka N. (2013). Immune cell infiltration as an indicator of the immune microenvironment of pancreatic cancer. Br. J. Cancer.

[108] De Monte L., Reni M., Tassi E., Clavenna D., Papa I., Recalde H., Braga M., Di Carlo V., Doglioni C., Protti M.P. (2011). Intratumor T helper type 2 cell infiltrate correlates with cancer-associated fibroblast thymic stromal lymphopoietin production and reduced survival in pancreatic cancer. J. Exp. Med..

[109] Prokopchuk O., Liu Y., Henne-Bruns D., Kornmann M. (2005). Interleukin-4 enhances proliferation of human pancreatic cancer cells: Evidence for autocrine and paracrine actions. Br. J. Cancer.

[110] Brunetto E., De Monte L., Balzano G., Camisa B., Laino V., Riba M., Heltai S., Bianchi M., Bordignon C., Falconi M. (2019). The IL-1/IL-1 receptor axis and tumor cell released inflammasome adaptor ASC are key regulators of TSLP secretion by cancer associated fibroblasts in pancreatic cancer. J. Immunother. Cancer.

[111] Protti M.P., De Monte L. (2012). Cross-talk within the tumor microenvironment mediates Th2-type inflammation in pancreatic cancer. Oncoimmunology.

[112] He S., Fei M., Wu Y., Zheng D., Wan D., Wang L., Li D. (2011). Distribution and Clinical Significance of Th17 Cells in the Tumor Microenvironment and Peripheral Blood of Pancreatic Cancer Patients. Int. J. Mol. Sci..

[113] McAllister F., Bailey J.M., Alsina J., Nirschl C.J., Sharma R., Fan H., Rattigan Y., Roeser J.C., Lankapalli R.H., Zhang H. (2014). Oncogenic Kras Activates a Hematopoietic-to-Epithelial IL-17 Signaling Axis in Preinvasive Pancreatic Neoplasia. Cancer Cell.

[114] Liyanage U.K., Moore T.T., Joo H.-G., Tanaka Y., Herrmann V., Doherty G., Drebin J.A., Strasberg S.M., Eberlein T.J., Goedegebuure P.S. (2002). Prevalence of Regulatory T Cells Is Increased in Peripheral Blood and Tumor Microenvironment of Patients with Pancreas or Breast Adenocarcinoma. J. Immunol..

[115] Hiraoka N., Onozato K., Kosuge T., Hirohashi S. (2006). Prevalence of FOXP3^+^ regulatory T cells increases during the progression of pancreatic ductal adenocarcinoma and its premalignant lesions. Clin. Cancer Res..

[116] Zhang Y., Lazarus J., Steele N.G., Yan W., Lee H.-J., Nwosu Z.C., Halbrook C.J., Menjivar R.E., Kemp S.B., Sirihorachai V.R. (2020). Regulatory T-cell Depletion Alters the Tumor Microenvironment and Accelerates Pancreatic Carcinogenesis. Cancer Discov..

[117] Orhan A., Vogelsang R.P., Andersen M.B., Madsen M.T., Hölmich E.R., Raskov H., Gögenur I. (2020). The prognostic value of tumour-infiltrating lymphocytes in pancreatic cancer: A systematic review and meta-analysis. Eur. J. Cancer.

[118] Brunet J., Denizot F., Luciani M., Roux-Dosseto M., Suzan M., Mattei M. (1987). A new member of the immunoglobulin superfamily-CTLA-4. Nature.

[119] Ishida Y., Agata Y., Shibahara K., Honjo T. (1992). Induced expression of PD-1, a novel member of the immunoglobulin gene superfamily, upon programmed cell death. EMBO J..

[120] Zou W., Chen L. (2008). Inhibitory B7-family molecules in the tumour microenvironment. Nat. Rev. Immunol..

[121] Leach D.R., Krummel M.F., Allison J.P. (1996). Enhancement of Antitumor Immunity by CTLA-4 Blockade. Science.

[122] Sunshine J., Taube J.M. (2015). PD-1/PD-L1 inhibitors. Curr. Opin. Pharmacol..

[123] Brahmer J.R., Tykodi S.S., Chow L.Q.M., Hwu W.-J., Topalian S.L., Hwu P., Drake C.G., Camacho L.H., Kauh J., Odunsi K. (2012). Safety and Activity of Anti–PD-L1 Antibody in Patients with Advanced Cancer. N. Engl. J. Med..

[124] Motzer R.J., Escudier B., McDermott D.F., George S., Hammers H.J., Srinivas S., Tykodi S.S., Sosman J.A., Procopio G., Plimack E.R. (2015). Nivolumab versus Everolimus in Advanced Renal-Cell Carcinoma. N. Engl. J. Med..

[125] Robert C., Long G.V., Brady B., Dutriaux C., Maio M., Mortier L., Hassel J.C., Rutkowski P., McNeil C., Kalinka-Warzocha E. (2015). Nivolumab in previously untreated melanoma without BRAF mutation. N. Engl. J. Med..

[126] Borghaei H., Paz-Ares L., Horn L., Spigel D.R., Steins M., Ready N.E., Chow L.Q., Vokes E.E., Felip E., Holgado E. (2015). Nivolumab versus Docetaxel in Advanced Nonsquamous Non–Small-Cell Lung Cancer. N. Engl. J. Med..

[127] Royal R.E., Levy C., Turner K., Mathur A., Hughes M., Kammula U.S., Sherry R.M., Topalian S.L., Yang J.C., Lowy I. (2010). Phase 2 trial of single agent Ipilimumab (anti-CTLA-4) for locally advanced or metastatic pancreatic adenocarcinoma. J. Immunother..

[128] Stromnes I.M., Hulbert A., Pierce R.H., Greenberg P.D., Hingorani S.R. (2017). T-cell localization, activation, and clonal expansion in human pancreatic ductal adenocarcinoma. Cancer Immunol. Res..

[129] Balli D., Rech A.J., Stanger B.Z., Vonderheide R.H. (2017). Immune cytolytic activity stratifies molecular subsets of human pancreatic cancer. Clin. Cancer Res..

[130] Alexandrov L.B., Nik-Zainal S., Wedge D.C., Aparicio S.A.J.R., Behjati S., Biankin A.V., Bignell G.R., Bolli N., Borg A., Børresen-Dale A.-L. (2013). Signatures of mutational processes in human cancer. Nature.

[131] Tumeh P.C., Harview C.L., Yearley J.H., Shintaku I.P., Taylor E.J.M., Robert L., Chmielowski B., Spasic M., Henry G., Ciobanu V. (2014). PD-1 blockade induces responses by inhibiting adaptive immune resistance. Nature.

[132] Rizvi N.A., Hellmann M.D., Snyder A., Kvistborg P., Makarov V., Havel J.J., Lee W., Yuan J., Wong P., Ho T.S. (2015). Mutational landscape determines sensitivity to PD-1 blockade in non-small cell lung cancer. Science.

[133] Humphris J.L., Patch A.M., Nones K., Bailey P.J., Johns A.L., McKay S., Chang D.K., Miller D.K., Pajic M., Kassahn K.S. (2017). Hypermutation In Pancreatic Cancer. Gastroenterology.

[134] Le D.T., Uram J.N., Wang H., Bartlett B.R., Kemberling H., Eyring A.D., Skora A.D., Luber B.S., Azad N.S., Laheru D. (2015). PD-1 Blockade in Tumors with Mismatch-Repair Deficiency. N. Engl. J. Med..

[135] Le D.T., Durham J.N., Smith K.N., Wang H., Bartlett B.R., Aulakh L.K., Lu S., Kemberling H., Wilt C., Luber B.S. (2017). Mismatch repair deficiency predicts response of solid tumors to PD-1 blockade. Science.

[136] Koikawa K., Kibe S., Suizu F., Sekino N., Kim N., Manz T.D., Pinch B.J., Akshinthala D., Verma A., Gaglia G. (2021). Targeting Pin1 renders pancreatic cancer eradicable by synergizing with immunochemotherapy. Cell.

[137] Beatty G.L., Chiorean E.G., Fishman M.P., Saboury B., Teitelbaum U.R., Sun W., Huhn R.D., Song W., Li D., Sharp L.L. (2011). CD40 Agonists Alter Tumor Stroma and Show Efficacy Against Pancreatic Carcinoma in Mice and Humans. Science.

[138] Mitchem J.B., Brennan D.J., Knolhoff B.L., Belt B.A., Zhu Y., Sanford D.E., Belaygorod L., Carpenter D., Collins L., Piwnica-Worms D. (2013). Targeting Tumor-Infiltrating Macrophages Decreases Tumor-Initiating Cells, Relieves Immunosuppression, and Improves Chemotherapeutic Responses. Cancer Res..

[139] Nywening T.M., Wang-Gillam A., Sanford D.E., Belt B.A., Panni R.Z., Cusworth B.M., Toriola A.T., Nieman R.K., Worley L.A., Yano M. (2016). Targeting tumour-associated macrophages with CCR2 inhibition in combination with FOLFIRINOX in patients with borderline resectable and locally advanced pancreatic cancer: A single-centre, open-label, dose-finding, non-randomised, phase 1b trial. Lancet. Oncol..

[140] Zhu Y., Knolhoff B.L., Meyer M.A., Nywening T.M., West B.L., Luo J., Wang-Gillam A., Goedegebuure S.P., Linehan D.C., De Nardo D.G. (2014). CSF1/CSF1R blockade reprograms tumor-infiltrating macrophages and improves response to T-cell checkpoint immunotherapy in pancreatic cancer models. Cancer Res..

[141] Wiehagen K.R., Girgis N.M., Yamada D.H., Smith A.A., Chan S.R., Grewal I.S., Quigley M., Verona R.I. (2017). Combination of CD40 agonism and CSF-1R blockade reconditions tumor-associated macrophages and drives potent antitumor immunity. Cancer Immunol. Res..

[142] Steele C.W., Karim S.A., Leach J.D.G., Bailey P., Upstill-Goddard R., Rishi L., Foth M., Bryson S., McDaid K., Wilson Z. (2016). CXCR2 Inhibition Profoundly Suppresses Metastases and Augments Immunotherapy in Pancreatic Ductal Adenocarcinoma. Cancer Cell.

[143] Kumar V., Donthireddy L., Marvel D., Condamine T., Wang F., Lavilla-Alonso S., Hashimoto A., Vonteddu P., Behera R., Goins M.A. (2017). Cancer-Associated Fibroblasts Neutralize the Anti-tumor Effect of CSF1 Receptor Blockade by Inducing PMN-MDSC Infiltration of Tumors. Cancer Cell.

[144] Lin M., Liang S., Wang X., Liang Y., Zhang M., Chen J., Niu L., Xu K. (2017). Percutaneous irreversible electroporation combined with allogeneic natural killer cell immunotherapy for patients with unresectable (stage III/IV) pancreatic cancer: A promising treatment. J. Cancer Res. Clin. Oncol..

[145] Gürlevik E., Fleischmann-Mundt B., Brooks J., Demir I.E., Steiger K., Ribback S., Yevsa T., Woller N., Kloos A., Ostroumov D. (2016). Administration of Gemcitabine after Pancreatic Tumor Resection in Mice Induces an Antitumor Immune Response Mediated by Natural Killer Cells. Gastroenterology.

[146] Manuel E.R., Chen J., D’Apuzzo M., Lampa M.G., Kaltcheva T.I., Thompson C.B., Ludwig T., Chung V., Diamond D.J. (2015). Salmonella-based therapy targeting indoleamine 2,3-dioxygenase coupled with enzymatic depletion of tumor hyaluronan induces complete regression of aggressive pancreatic tumors. Cancer Immunol. Res..

[147] Le D.T., Wang-Gillam A., Picozzi V., Greten T.F., Crocenzi T., Springett G., Morse M., Zeh H., Cohen D., Fine R.L. (2015). Safety and survival with GVAX pancreas prime and Listeria monocytogenes-expressing mesothelin (CRS-207) boost vaccines for metastatic pancreatic cancer. J. Clin. Oncol..

[148] Bernhardt S.L., Gjertsen M.K., Trachsel S., Møller M., Eriksen J.A., Meo M., Buanes T., Gaudernack G. (2006). Telomerase peptide vaccination of patients with non-resectable pancreatic cancer: A dose escalating phase I/II study. Br. J. Cancer.

[149] Mayanagi S., Kitago M., Sakurai T., Matsuda T., Fujita T., Higuchi H., Taguchi J., Takeuchi H., Itano O., Aiura K. (2015). Phase I pilot study of Wilms tumor gene 1 peptide-pulsed dendritic cell vaccination combined with gemcitabine in pancreatic cancer. Cancer Sci..

[150] Lutz E.R., Wu A.A., Bigelow E., Sharma R., Mo G., Soares K., Solt S., Dorman A., Wamwea A., Yager A. (2014). Immunotherapy converts nonimmunogenic pancreatic tumors into immunogenic foci of immune regulation. Cancer Immunol. Res..

[151] Keenan B.P., Saenger Y., Kafrouni M.I., Leubner A., Lauer P., Maitra A., Rucki A.A., Gunderson A.J., Coussens L.M., Brockstedt D.G. (2014). A listeria vaccine and depletion of t-regulatory cells activate immunity against early stage pancreatic intraepithelial neoplasms and prolong survival of mice. Gastroenterology.

[152] Thind K., Padrnos L.J., Ramanathan R.K., Borad M.J. (2017). Immunotherapy in pancreatic cancer treatment: A new frontier. Therap. Adv. Gastroenterol..

[153] Aung K.L., Fischer S.E., Denroche R.E., Jang G.-H., Dodd A., Creighton S., Southwood B., Liang S.-B., Chadwick D., Zhang A. (2018). Genomics-Driven Precision Medicine for Advanced Pancreatic Cancer: Early Results from the COMPASS Trial. Clin. Cancer Res..

[154] Holter S., Borgida A., Dodd A., Grant R., Semotiuk K., Hedley D., Dhani N., Narod S., Akbari M., Moore M. (2015). Germline BRCA Mutations in a Large Clinic-Based Cohort of Patients with Pancreatic Adenocarcinoma. J. Clin. Oncol..

[155] Golan T., Kindler H.L., Park J.O., Reni M., Mercade T.M., Hammel P., Van Cutsem E., Arnold D., Hochhauser D., Locker G.Y. (2018). Geographic and ethnic heterogeneity in the BRCA1/2 pre-screening population for the randomized phase III POLO study of olaparib maintenance in metastatic pancreatic cancer (mPC). J. Clin. Oncol..

[156] O’Connor M.J. (2015). Targeting the DNA Damage Response in Cancer. Mol. Cell.

[157] Golan T., Hammel P., Reni M., Van Cutsem E., Macarulla T., Hall M.J., Park J.-O., Hochhauser D., Arnold D., Oh D.-Y. (2019). Maintenance Olaparib for Germline BRCA-Mutated Metastatic Pancreatic Cancer. N. Engl. J. Med..

[158] Romero-Calvo I., Weber C.R., Ray M., Brown M., Kirby K., Nandi R.K., Long T.M., Sparrow S.M., Ugolkov A., Qiang W. (2019). Human Organoids Share Structural and Genetic Features with Primary Pancreatic Adenocarcinoma Tumors. Mol. Cancer Res..

[159] Frappart P.-O., Hofmann T.G. (2020). Pancreatic Ductal Adenocarcinoma (PDAC) Organoids: The Shining Light at the End of the Tunnel for Drug Response Prediction and Personalized Medicine. Cancers.

